# Can a multidisciplinary approach slow renal progression in CKD patients?

**DOI:** 10.7150/ijms.53189

**Published:** 2021-03-03

**Authors:** Ampornpan Theeranut, Nonglak Methakanjanasak, Pattama Surit, Junto Srina, Dhavee Sirivongs, Doangjai Adisuksodsai, Sunee Lertsinudom, Kittisak Sawanyawisuth

**Affiliations:** 1 Department of Adult Nursing, Faculty of Nursing, Khon Kaen University, Khon Kaen, 40002 Thailand.; 2 Research and Training Center for Enhancing Quality of Life of Working Age People, Khon Kaen University, Khon Kaen, Thailand.; 3 Department of Medicine, Chumpae Hospital, Khon Kaen, 40002Thailand.; 4 Department of Clinical Pharmacy, Faculty of Pharmaceutical Sciences, Khon Kaen University, Khon Kaen, 40002 Thailand.; 5 Department of Medicine, Faculty of Medicine, Khon Kaen University, Khon Kaen, 40002 Thailand.

**Keywords:** CKD, education, glomerular filtration rate.

## Abstract

**Background**: Several randomized controlled trials have examined the benefits of multidisciplinary CKD care on estimated glomerular filtration rate (eGFR). But, the results are inconclusive.

**Purpose:** This study aimed to evaluate whether or not multidisciplinary CKD care was beneficial in terms of CKD progression.

**Methods**: This is a randomized controlled trial and conducted at community hospital, Thailand. The inclusion criteria were patients with age of 18 years or older and diagnosed with up to stage 3b CKD based on the KDIGO guidelines. Eligible patients divided into two groups: intervention and control group. The intervention group received a type of multidisciplinary treatment, while patients in the control group received the standard treatment administered at the outpatient clinic. The primary outcome was eGFR outcomes at three months after enrollment.

**Results**: During the study period, there were 334 patients who met the study criteria. Eligible patients were divided into two groups: intervention (166 patients; 49.70%) and control (168 patients; 50.30%). There were three outcomes that differed significantly between the two groups at 3 months: mean difference of eGFR from baseline, proportion of patients with eGFR decline greater than 4 mL/min/1.73 m^2^, and difference in CKD stage from baseline. A significantly higher percentage of patients in the intervention group experienced CKD improvement by one stage (24.10% vs 5.95%), and a significantly lower percentage experienced decline by one stage (8.43% vs 35.12%) than in the control group.

**Conclusion**: Slower renal progression in patients with up to stage 3b CKD was shown in patients who were treated by a multidisciplinary approach.

## Introduction

Chronic kidney disease **(**CKD**)** is defined as the presence of albuminuria greater than or equal to 30 mg**/**day or an estimated glomerular filtration rate (eGFR) of less than 60 mL**/**min**/**1**.**73 m^2^ for more than three months regardless of cause 1**.** The condition is a known cardiovascular risk factor**.** A study found that patients with eGFR below 60 ml**/**min**/**1**.**73m^2^ had a 13**%** higher risk of cardiovascular death 2**.** Additionally, CKD patients require renal replacement therapy at a rate of approximately 1**.**6 per 100 patient**-**years 3**.** Interventions are required in order to reduce both cardiovascular risk and renal progression**.**

Reversible causes of renal failure, such as hypovolemia and NSAID use, should generally be avoided**.** Additionally, ACEI**/**ARB may slow renal progression in particular situations 1**.** Statin therapy has been shown to prevent major cardiovascular events by 20**%** with 1 mmol**/**L of LDL**-**c reduction 4**.** Other possible treatments include protein restriction, smoking cessation, and blood pressure**/**glucose control**.** Due to the variety of treatments required for CKD, a multidisciplinary approach may be needed 5**.** Additionally, the incidence of CKD is increasing leading to a need to improve primary healthcare system by the multidisciplinary approach 6.

Previous studies have shown that cost at dialysis initiation and risk of death were lower at multidisciplinary CKD clinics than usual care at 942 USD vs 2410 USD and hazard ratio of 2**.**17, respectively 7, 8**.** Dialysis initiation and mortality were significantly lower in multidisciplinary treatment group than the control (13.9% vs 43%; p < 0.001 and 1.7% vs 10.1%; p < 0.001) as well as medical cost reduction 9, 10. However, there are still conflicting data on multidisciplinary management on eGFR. Two studies showed improvement of eGFR by multidisciplinary management by 2.74-13.39 ml/min 11, 12, while a systematic review and two randomized controlled trials did not 13-15. This study aimed at adding a study on evaluating whether or not multidisciplinary CKD care was beneficial in terms of CKD progression**.**

## Methods

This was an intervention study conducted at Chumpae Hospital in Khon Kaen, Thailand. This hospital is a large community hospital with 150 beds and serves a population of 123,739 people**.** The inclusion criteria were patients with age of 18 years or older and diagnosed with up to stage 3b CKD based on the KDIGO guidelines**.** The exclusion criteria were those end stage renal disease hospitalized and required dialysis, unable to participate the study toward the end, or had co-morbid diseases that needed further treatment such as cancer, HIV infection, or systemic lupus erythromatosus. The study period was between January 2017 and April 2018**.** Eligible patients were randomly assigned to either the intervention or control group using a simple random sampling method**.**

The intervention group received a type of multidisciplinary treatment called the Chumpae model for delaying dialysis in CKD patients**.** This model is an intervention based on the chronic care model and consists of four principle systems, as follows**:** delivery, clinical information, decision support, and self**-**management support**.** The delivery system is a healthcare system for individual patients in which treatment is administered by internists, registered nurses, and pharmacists**.** For complicated cases, specialized nurses are assigned to care for individual patients based on social and cultural factors**.** The clinical information system provides updated, systematic clinical evidence for the patients and care team**.** The decision support system encourages decision making through the exchange of ideas among patients**.** Finally, the self**-**management support system empowers patients by employing the specialized nurses and expert patients to facilitate self**-**monitoring and self**-**efficacy**.**

The procedures were performed in an outpatient setting with six steps**:** 1**)** clinical information evaluation and decision support by specialized nurse 2**)** self**-**management support by expert patients and pharmacist 3**)** decision support by internists 4**)** appointment and follow**-**up by specialized nurse 5**)** medications by pharmacist and 6**)** self**-**management support by patients and caregivers**.** The specialized nurse also contacted patients by phone regarding any concerns or issues they had while at home**.** The media used in the intervention included a log book, a video regarding CKD care, a CKD pamphlet, and advice from the expert patients**.** Patients in the control group received the standard treatment administered at the outpatient clinic**.** The CKD treatment plans for patients in both groups were decided on by an attending physician**.** Baseline characteristics and clinical variables were assessed**.** The eGFR outcomes of eligible patients were then evaluated at three months**.**

### Sample size calculation

As previously reported 11, the mean difference of eGFR between the multidisciplinary management and non- multidisciplinary management group was 13.39 ml/min. For a power of 90% and confidence of 99%, the sample size in each arm was 148. With a 10% drop out rate, the required of study population was 163 patients.

### Statistical analysis

Data were reported as mean (SD) for numerical variables and number (percentage) for categorical variables. Comparisons between groups were computed by using descriptive statistics at both baseline and three months**.** An unpaired t-test was used for a comparison of numerical variables with normal distribution, while Chi- square test or Fisher Exact test was used when appropriate for a comparison of categorical variables. All statistical analysis was conducted using STATA software, version 10**.**1 **(**College Station, Texas, USA**).** The study protocol was approved by the institutional review board, Khon Kaen University, Thailand (HE602078).

## Results

During the study period, there were 334 patients who met the study criteria**.** Eligible patients were divided into two groups**:** intervention **(**166 patients; 49**.**70**%)** and control** (**168 patients; 50**.**30**%).** Five of the studied variables were non**-**significant including sex, religion, and proportions of patients with dyslipidemia, gout, and coronary artery disease **(**Table 1**).** The control group had significantly higher income than the intervention group **(**5585 vs 2516 baht**/**month; p value < 0**.**001**)** as well as education levels, co-morbid diseases, and previous experiences on CKD (Table 1)**.** In terms of physical signs, the intervention group had significantly higher waist circumference **(**91**.**12 vs 76**.**73 cm**)** but lower diastolic blood pressure **(**72**.**66 vs 74**.**82 mmHg**)** than the control group **(**Table 2**).**

At baseline, the intervention group had significantly lower eGFR than the control group but the eGFR was higher at 3 months in the intervention group **(**48**.**77 vs 47**.**89 ml**/**min**/**1**.**73m^2^; p value = 0**.**395**).** There were three outcomes that differed significantly between the two groups at 3 months**:** mean difference of eGFR from baseline, proportion of patients with eGFR decline greater than 4 mL**/**min**/**1**.**73 m^2^, and difference in CKD stage from baseline **(**Table 3**).** A significantly higher percentage of patients in the intervention group experienced CKD improvement by one stage **(**24**.**10**%** vs 5**.**95**%)**, and a significantly lower percentage experienced decline by one stage **(**8**.**43**%** vs 35**.**12**%)** than in the control group**.**

## Discussion

This randomized control trial showed the benefits of employing a multidisciplinary approach in the treatment of CKD patients**.** The multidisciplinary treatment for up to stage 3b CKD slowed CKD progression as evidenced by improvement in eGFR (**+**1**.**76 vs **-**5**.**85 ml**/**min**/**1**.**73m^2^) and the lower proportion of patients with eGFR decline > 4 ml**/**min**/**1**.**73m^2^**)** and led to improvements in CKD stage as shown in Table 3**.** These benefits were statistically significant despite patients in the intervention group having more severe CKD at baseline**.** Baseline eGFR was significantly lower in the intervention group than the control group **(**47**.**01 vs 53**.**75 ml**/**min**/**1**.**73m^2^; p value < 0**.**001**)**. And, there were higher proportions of patients with stage 3a **(**59**.**04**%** vs 48**.**21**%)** and 3b **(**38**.**55**%** vs 22**.**62**%)** CKD despite the use of a simple random sampling method**.** These differences highlight the benefits of the multidisciplinary approach**.**

A previous randomized controlled study showed that composite end**-**points, including eGFR, significantly improved after a self**-**management program that involved diet and exercise, but the difference in eGFR alone was not significant **(**1**.**2**%** vs 11**.**2**%**; p value = 0**.**209**)** 16**.** However, that study differed from the present study in some key respects**.** First, while the previous study enrolled patients with stage 3 and 4 CKD, this study only enrolled patients with up to stage 3b, which may have resulted in better outcomes**.** Second, the previous study only examined the self**-**management support system, while our study also implemented a delivery system, clinical information system, and decision support system**.** The specialized nurse and expert patients also played important roles in CKD care**.** A previous study found that the involvement of a single experienced renal nurse practitioner led to greater improvements in various renal surrogate outcomes, such as hemoglobin** (**11**.**6 vs 10**.**8 g**/**dL, p value < 0.05**)** and serum albumin **(**3**.**4 vs 3**.**0 g**/**dL; p value < 0.01**)**, compared with care provided by multiple nephrology trainees 17**.** As was the case in this study, the renal nurse was better able to encourage the patients to stick to the treatment plan**.**

The strength of this study is that it was conducted as a randomized controlled trial and found benefits with regard to renal progression**.** As mentioned earlier, the benefits of multidisciplinary management in CKD are simple and cheap and it can save costs on healthcare, delay dialysis initiation, and reduce mortality 17. This multidisciplinary management model is feasible in community hospitals. The team comprised of simple combination of internists, registered nurses, and pharmacists. These medical personnel are available in all levels of hospitals. Training of patients, special nurses, and caregivers are also required.

There were some limitations**.** First, the results of this study may only apply to community hospital settings and patients with CKD stage 3b or lower**.** Further studies may be needed to confirm these results in other more complicated setting such as tertiary care hospitals 18-20**.** Second, as the primary outcomes were measured at three months, longer term evaluation may be necessary**.** Third, some factors associated with CKD treatment were not studied such as statin therapy or toxic substances 21-23**.** However, the randomization process may have negated these issues**.** Fourth, the adherence rate was not evaluated**.** However, the benefits found may be significant regardless of adherence rate**.** Finally, there were some different factors at baseline despite randomized method. The outcomes of the study may be affected by these differences. For example, the intervention group had lower education level than the control group but better outcomes. These results may imply that education level may not affect the results of this multidisciplinary approach. Further studies are needed to confirm the results of this study.

In conclusion, this randomized controlled trial showed slower renal progression in patients with up to stage 3b CKD who were treated using a multidisciplinary approach**.**

## Figures and Tables

**Table 1:** Baseline characteristics of patients with chronic kidney disease (CKD) who received an intervention for delaying dialysis (intervention group) compared to controls.

Factors	Intervention group (n = 166)	Control group (n = 168)	p value
Age, years	66**.**06 **(**7**.**94**)**	68**.**66 **(**8**.**93**)**	0**.**005
Male sex	64 **(**38**.**55**)**	64** (**38**.**10**)**	0**.**999
Marital status			0**.**033
Single	4 **(**2**.**42**)**	5 **(**3**.**07**)**	
Married	130 **(**78**.**79**)**	143 **(**87**.**73**)**	
Widowed	30 **(**18**.**18**)**	13 **(**7**.**98**)**	
Divorced**/**separated	1 **(**0**.**61**)**	2 **(**1**.**23**)**	
Religion**:** Buddhism	165 **(**99**.**40**)**	168** (**100**.**0**)**	0**.**315
Highest education			< 0**.**001
No	8 **(**4**.**91**)**	20 **(**12**.**20**)**	
Primary school	147 **(**90**.**18**)**	80 **(**48**.**78**)**	
Secondary school	6 **(**3**.**68**)**	45 **(**27**.**44**)**	
College or higher	2 **(**1**.**23**)**	13 **(**7**.**93**)**	
Occupation			< 0**.**001
Unemployed	89 **(**53**.**61**)**	51 **(**30**.**54**)**	
Agriculturist	57 **(**34**.**34**)**	34 **(**20**.**36**)**	
Employee	12 **(**7**.**23**)**	61 **(**36**.**53**)**	
Government officer	2 **(**1**.**20**)**	18 **(**10**.**78**)**	
Mean **(**SD**)** income, Baht	2516**.**77 **(**4163**.**34**)**	5585**.**35 **(**5566**.**83**)**	< 0**.**001
Primary caregiver			0**.**038
Spouse	71 **(**42**.**77**)**	48 **(**32**.**43**)**	
Children	90 **(**54**.**22**)**	89 **(**60**.**14**)**	
Siblings	2 **(**1**.**20**)**	10 **(**6**.**76**)**	
Cousin**/**relative	2 **(**1**.**20**)**	1 **(**0**.**68**)**	
Previous experience with nutrition in CKD	40 **(**24**.**10**)**	56 **(**57**.**73**)**	< 0**.**001
Previous experience with safety drugs in CKD	27 **(**16**.**27**)**	57 **(**58**.**76**)**	< 0**.**001
Previous experience with deteriorating renal function	12 **(**7**.**27**)**	41 **(**42**.**27**)**	< 0**.**001
Co**-**morbid diseases			
Hypertension	152 **(**91**.**57**)**	138 **(**82**.**63**)**	0**.**021
Diabetes	120 **(**72**.**29**)**	156 **(**93**.**41**)**	< 0**.**001
Dyslipidemia	76 **(**45**.**78**)**	74 **(**44**.**31**)**	0**.**826
Gout	14 **(**8**.**43**)**	9 **(**5**.**39**)**	0**.**289
Coronary artery disease	6 **(**3**.**61**)**	1 **(**0**.**60**)**	0**.**067

Note. Data presented as number (percentage) unless indicated otherwise; data for the intervention and control group may not be equal to 166 and 168 due to missing data.

**Table 2:** Physical factors and laboratory results of patients with chronic kidney disease (CKD) who received an intervention for delaying dialysis (intervention group) compared to controls.

Factors	Intervention group (n = 166)	Control group (n = 168)	p value
Body mass index, kg**/**m2	25**.**33 **(**3**.**94**)**	25**.**06 **(**4**.**10**)**	0**.**564
Waist, cm	91**.**12 **(**9**.**94**)**	76**.**73 **(**19**.**08**)**	< 0**.**001
Systolic blood pressure, mmHg	135**.**36 **(**15**.**30**)**	138**.**41 **(**17**.**03**)**	0**.**087
Diastolic blood pressure, mmHg	72**.**66 **(**8**.**64**)**	74**.**82 **(**9**.**15**)**	0**.**027
HbA1C, **%**	7**.**82 **(**2**.**07**)**	8**.**02 **(**2**.**05**)**	0**.**419
LDL**-**c, mg**/**dL	103**.**87** (**36**.**18**)**	97**.**69 **(**40**.**33**)**	0**.**142
HDL**-**c, mg**/**dL	44**.**40 **(**13**.**06**)**	42**.**63 **(**13**.**02**)**	0**.**215

Note. Data presented as mean (SD).

**Table 3:** Glomerular filtration rate (eGFR) and staging of patients with chronic kidney disease (CKD) who received an intervention for delaying dialysis (intervention group) compared to controls at baseline and after 3 months of intervention.

Factors	Intervention group (n = 166)	Control group (n = 168)	p value
Baseline mean **(**SD**)** of eGFR	47**.**01 **(**8**.**31**)**	53**.**75 **(**11**.**74**)**	< 0**.**001
Mean (SD) eGFR at 3 months	48**.**77 **(**10**.**08**)**	47**.**89 **(**8**.**85**)**	0**.**395
Mean **(**SD**)** differences of eGFR from baseline	**+**1**.**76 **(**7**.**51**)**	**-**5**.**85 **(**9**.**03**)**	< 0**.**001
Decline of eGFR > 4 mL**/**min**/**1**.**73 m2 after 3 months, n	27 **(**16**.**27**)**	90 **(**53**.**57**)**	< 0**.**001
Baseline CKD staging			< 0**.**001
2	4 **(**2**.**41**)**	49 **(**29**.**17**)**	
3a	98 **(**59**.**04**)**	81 **(**48**.**21**)**	
3b	64 **(**38**.**55**)**	38 **(**22**.**62**)**	
CKD staging at 3 months			0**.**124
2	20 **(**12**.**05**)**	16 **(**9**.**52**)**	
3a	96 **(**57**.**83**)**	94 **(**55**.**95**)**	
3b	46 **(**27**.**71**)**	58 **(**34**.**52**)**	
4	4 **(**2**.**41**)**	0	
Differences of CKD stage from baseline			< 0**.**001
Improved by one stage	40 **(**24**.**10**)**	10 **(**5**.**95**)**	
No progression	112 **(**67**.**47**)**	97 **(**57**.**74**)**	
Declined by one stage	14 **(**8**.**43**)**	59 **(**35**.**12**)**	
Declined by two stages	0	2 **(**1**.**19**)**	

Note. Data presented as number (percentage) unless indicated otherwise.
